# Advances in Catalysts for Urea Electrosynthesis Utilizing CO_2_ and Nitrogenous Materials: A Mechanistic Perspective

**DOI:** 10.3390/ma17092142

**Published:** 2024-05-03

**Authors:** Mengfei Zhang, Tianjian Feng, Xuanming Che, Yuhan Wang, Pengxian Wang, Mao Chai, Menglei Yuan

**Affiliations:** 1 Queen Mary University of London Engineering School, Northwestern Polytechnical University, Xi’an 710129, China; 2 Guoneng Shanxi Hequ Power Generation Co., Ltd., Xinzhou 036500, China; 3 State Key Laboratory of Solidification Processing, Center for Nano Energy Materials, School of Materials Science and Engineering, Northwestern Polytechnical University, Xi’an 710072, China

**Keywords:** electrocatalytic, urea electrocatalysis, catalyst design, microscopic mechanisms

## Abstract

Electrocatalytic urea synthesis from CO_2_ and nitrogenous substances represents an essential advance for the chemical industry, enabling the efficient utilization of resources and promoting sustainable development. However, the development of electrocatalytic urea synthesis has been severely limited by weak chemisorption, poor activation and difficulties in C–N coupling reactions. In this review, catalysts and corresponding reaction mechanisms in the emerging fields of bimetallic catalysts, MXenes, frustrated Lewis acid–base pairs and heterostructures are summarized in terms of the two central mechanisms of molecule–catalyst interactions as well as chemical bond cleavage and directional coupling, which provide new perspectives for improving the efficiency of electrocatalytic synthesis of urea. This review provides valuable insights to elucidate potential electrocatalytic mechanisms.

## 1. Introduction

Carbon dioxide (CO_2_) dominates as a greenhouse gas and the most common source of carbon in the atmosphere, while nitrogen (N_2_) accounts for 78% of the total the atmosphere [1,2]. The utilization of carbon and nitrogen sources in the atmosphere to produce high-value and multifunctional chemicals has become a major goal due to their relative ease of availability. In addition to being a common nitrogen fertilizer, urea is also the essential organic raw material for the composition of urea–formaldehyde, urea–melamine and barbiturate resins, which dominates the modern pharmaceutical and agricultural industries [3,4,5]. However, the high stability of triple bond in N_2_ (N≡N) and double bond in CO_2_ (C=O) result in daunting challenges facing the production of urea. Despite thermal catalytic technology readily overcoming the aforementioned dilemma, it usually results in significant energy consumption and environmental impact [6,7].

Therefore, new urea synthesis strategies with the efficient utilization of renewable energy sources which meet the dual needs of energy and atomic economy are required [8,9]. Of the methods for green urea production, the electrocatalytic synthesis of urea using CO_2_ and N_2_ at lower cost and under mild conditions is highly anticipated [10,11,12,13]. The improvement of the efficiency of urea production by overcoming the challenges of electrocatalytic urea synthesis has been a subject of intense interest [14]. At present, major challenges in electrocatalytic synthesis of urea include (i) highly stable chemical bond of N_2_ and CO_2_, which requires specific catalyst for activation, (ii) low solubility of reactant molecular in water electrolyte, and (iii) parallel competitive reduction reactions resulting in low selectivity [1,4,15,16,17]. 

To address the above challenges in the electrocatalytic synthesis of urea, this non-systematic review focuses on the principles and effects of novel bimetallic catalysts, heterogeneous interface-rich catalysts, and frustrated Lewis pairs based on the dual mechanisms of molecule–catalyst interactions as well as chemical bond cleavage and directional coupling. In order to improve the Faraday efficiency (FE) and atomic efficiency (AE) of urea synthesis, the design principles of electrocatalysts such as increasing the number of active sites, increasing the specific surface area and varying the peripheral electron density have been comprehensively analyzed [3,7,8,18,19,20]. This review provides important implications for advancing the industrial application of urea electrosynthesis technology.

## 2. Molecular Catalyst Interaction Mechanism Achieving Reactant Targeted Adsorption

During the electrochemical synthesis of urea, improving the FE and AE of CO_2_ and N_2_ represents a significant challenge due to the high dissociation energy of chemical bonds and the limited solubility of reactant molecules in water [10,11,12,21,22]. A great body of work has already sought to address many of these limitations like exploring novel synthesis strategies to enhance the specific adsorption efficiency of reactants by introducing new active sites and optimizing catalyst morphology and structure to achieve superior catalytic performance [16,17,19,22,23,24,25,26]. Researchers have optimized the structure of catalyst by fabricating porous electrodes, e.g., designing nanocrystalline materials, MXenes, to increase specific surface areas and active sites [19], thereby increasing the binding sites between the catalyst and reactants [23,24]. Moreover, the rationally designed catalysts such as metals and their oxides, composite materials and peroxide-mixed materials have been utilized to enhance the specific adsorption of CO_2_ and N_2_ [22,25]. Recently, researchers have also proposed new catalysts such as heterojunctions and perovskite hybrids to alter spatial charge distributions, driving the specific adsorption of reactants and catalysts [8,15]. In summary, these advancements have paved the way for large-scale green urea production.

### 2.1. Bimetallic Catalyst

#### 2.1.1. Double Transition-Metal MXenes

MXenes are a type of 2D-layered transition metal carbides or nitrides [27]. They are broadly applicable in multiple areas of chemistry, due to their variable electronic structure, large specific surface area and numerous exposed active sites. Double transition-metal MXenes are important for the electrocatalytic C–N conjugation of urea [18,28,29,30,31,32]. 

**Figure 1:**
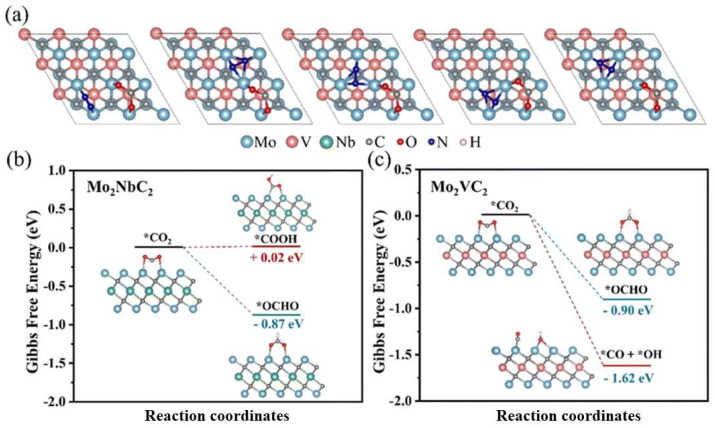
(**a**) Possible cover sites for N_2_ and CO_2_ on Mo_2_VC_2_ and the changes in (**b**) Mo_2_NbC_2_ and (**c**) Mo_2_VC_2_ with CO_2_ covered after the first H was added at different sites. Reproduced with permission from Ref. [32]. (* represent the active sites) Copyright 2023 Royal Society of Chemistry.

The related chemical formula is M_n+1_X_n_ (M is a transition metal and X is C and/or N, n = 1, 2, 3), and investigations have shown that the electronic structure of MXene catalysts gives rise to an level of selectivity and catalytic activity in catalyzing urea synthesis reactions when n = 2. The reactant molecules CO_2_ and N_2_ were adsorbed on the Mo_2_VC_2_ surface, and based on the face center cubic (fcc) sites of CO_2_ adsorption shown in Figure 1a, the adsorption energies represented by different structures were calculated; comparing the energy reduction values for reactant adsorption shows that Mo and V have a mutually promoting effect on the adsorption of reactant molecules. Furthermore, the corresponding adsorption energies shown in Figure 1b,c were compared, and the decrease in adsorption energy on Mo_2_VC_2_ is more obvious when the first H is added to CO_2_, which is due to the unique electronic structure of Mo_2_VC_2_ being able to provide more electrons for *CO_2_, making the breakage of the C-O bond easier; therefore, the route of Mo_2_VC_2_ is the primary route for urea synthesis. It was noteworthy that the projected density of states (PDOS) calculation further revealed that the outer atoms of unique ordered MXenes (transition metal atoms in the outer layer, and other metals in the inner layer) possessed a higher d-band center compared to pristine Mo_3_C_2_ and Ti_3_C_2_ samples. It was further demonstrated that the novel MXene may increase the d-band energy of metal sites. The downward shift of d-band center favored reactant adsorption onto the catalyst; in other words, it decreased the absorption energy of inert gas molecules to the surface of MXenes [32].

#### 2.1.2. Pd–Cu Bimetallic Catalyst

Bimetallic catalysts are composed of two different metal elements, which usually exhibited higher catalytic activity and selectivity than single metal catalysts due to the unique synergistic interaction between different metals. In bimetallic catalysts, two metal elements can interact with each other in different ways, giving bimetallic catalysts more reaction mechanisms and a wider range of applications [33,34,35].

**Figure 2:**
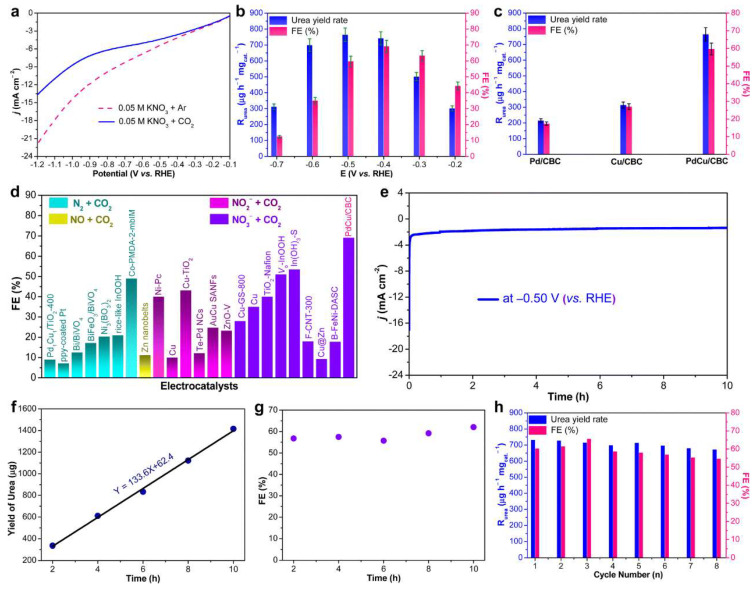
(**a**) LSV curves of the PdCu/CBC in 0.05 M KNO_3_ electrolyte with Ar or CO_2_ feeding gas. (**b**) Dependence of *R*urea and FE on the applied potentials. (**c**) *R*urea and FE of the PdCu/CBC, Cu/CBC and Pd/CBC at −0.50 V (vs. RHE) for 2 h reaction. (**d**) Comparison of the FEs of the reported electrocatalysts and the PdCu/CBC with different nitrogen sources. (**e**) Stability test of the PdCu/CBC at −0.50 V (vs. RHE). (**f**) The urea yield and (**g**) FE for urea production over the PdCu/CBC toward electrochemical coupling NO^3−^ with CO_2_ with reaction time at −0.50 V (vs. RHE). (**h**) Recycling stability test of the PdCu/CBC. Reproduced with permission from Ref. [36]. Copyright 2023 Royal Society of Chemistry.

Regarding the PdCu/CBC bimetallic catalyst, the Pd- and Cu-alloyed nanoparticles were dispersed in carbonized bacterial cellulose (CBC) and utilized for urea electrosynthesis [36], and CO_2_ can be specifically adsorbed onto the porous surface of the catalyst on account of the excellent coordination ability of oxygen-rich functional groups in CBC with metal ions and the rich active sites of the bimetallic alloy for binding CO_2_ [37,38]. Despite the Cu thin films possessing superior selectivity in terms of converting CO_2_ to HCOO-, the related intermediates cannot be detected by in situ spectroscopy [39]. The adsorption of more carbon dioxide can also suppress the formation of side reactions. Figure 2a displays the linear sweep voltammetry (LSV) curve of PdCu/CBC in an CO_2_- or Ar-saturated 0.05 M KNO_3_ electrolyte. It can be observed that the participation of CO_2_ resulted in an increase in current density compared to that in the Ar-saturated electrolyte. This was attributed to the effective suppression of the NO_3_^−^RR and/or HER by CO_2_ at a specific voltage, thus promoting the C–N coupling for urea synthesis. The carbonization process of PdCu/CBC will, in situ, convert the chemically impregnated Pd^2+^ and Cu^2+^ into a nano-alloy that is anchored to the carbon carrier by the M-O coordination configuration, further engendering the excellent catalytic stability and the high FE of the catalyst. In the experiment, after specifically investigating the urea yield rate and Faraday efficiency under different potentials (Figure 2b), it was confirmed that PdCu/CBC exhibited superior electrocatalytic activity for urea synthesis. To demonstrate the superiority of PdCu/CBC more rigorously, the urea synthesis yield and FE of single-metal Pd/CBC and Cu/CBC electrocatalysts were also evaluated (Figure 2c). Additionally, when comparing the recently reported urea electrosynthesis catalysts with the as-prepared PdCu/CBC, the latter also yielded a higher urea yield and FE. Figure 2e–h further demonstrated the excellent stability of PdCu/CBC catalysts. During the electrolysis of CO_2_-saturated 0.05 M KNO_3_ at −0.50 V (vs. RHE) for 10 h, it was found that the changes in current density were almost negligible throughout the testing period (Figure 2e). Meanwhile, there was a good linear relationship between urea yield and reaction time (Figure 2f), and the corresponding changes in FE were also minimal (Figure 2g). In further cyclic stability tests, only minor changes were observed in the urea yield and FE after eight consecutive cycles (Figure 2h). Therefore, the stability of PdCu/CBC was undoubtedly excellent. Moreover, the Pd and Cu sites in the PdCu alloy were proven to be catalytically active centers for CO_2_ adsorption and activation by subsequent high-temperature desorption experiments [36].

### 2.2. Heterogeneous Interface-Rich Catalysts

#### 2.2.1. Perovskite Hybrids BiFeO_3_/BiVO_4_

Electronic states on the electrocatalyst surface are crucial for adsorbing gas molecules and subsequent coupling C–N bonds. Perovskite-structured transition-metal oxide semiconductors (ABO_3_) acquire a unique electronic structure that alters electron density when combined with other domains [40,41]. In addition, perovskite heterostructures will drive localized charge redistribution through band bending at the heterojunction interface, enabling selective adsorption and activation of small molecules. Therefore, creating built-in electric fields to enhance charge redistribution in perovskite heterostructures is a promising strategy [8]. Theoretical calculations suggested that BiFeO_3_/BiVO_4_ hybrids with hetero structured perovskites possess a strong integrated electric field that can facilitate the redistribution of charge in close proximity to one another. Accordingly, the electrophilic nature of CO_2_ and the nucleophilic nature of N_2_ specifically adsorb and activate them on newly constructed internal electric fields.

**Figure 3:**
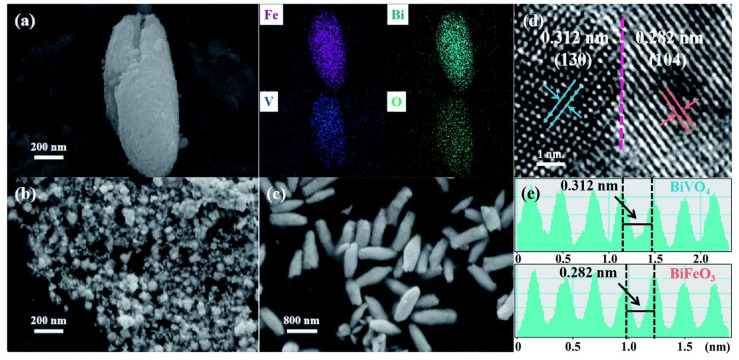
(**a**) SEM image and the corresponding elemental mapping of BiFeO_3_/BiVO_4_ hybrids; SEM images of (**b**) BiFeO_3_ and (**c**) BiVO_4_; (**d**) high-resolution TEM image of BiFeO_3_/BiVO_4_ hybrids, and the dotted line represents the heterointerfaces; (**e**) the well-resolved lattice fringe of BiFeO_3_/BiVO_4_ hybrids. Reproduced with permission from Ref. [15]. (blue color represent BiVO_4_, while red color represent BiFeO_3_) Copyright 2021 Royal Society of Chemistry.

As determined by density functional theory (DFT), the adsorption of both N_2_ and CO_2_ on novel BiFeO_3_/BiVO_4_ perovskite structures can be significantly reduced, indicating that the catalyst displayed high adsorption capacity for the two gas molecules, resolving a major issue in the reaction [15]. The BiFeO_3_/BiVO_4_ perovskite structure was designed and synthesized using a facile ultrasonic bath method. As shown in Figure 3a, the field emission scanning electron microscopy (FE-SEM) image revealed that the BiFeO_3_/BiVO_4_ catalysts possessed a rice-like morphology, with an average length of 1 μm and an average diameter of approximately 400 nm. The corresponding elemental mapping images indicated the uniform distribution of Bi, Fe, V and O elements. Intriguingly, the pristine BiVO_4_ maintains the same morphology as the heterostructure hybrid (Figure 3c), while BiFeO_3_ presents an irregular nanoparticle structure (Figure 3b). In Figure 3d, the well-resolved lattice fringes of 0.282 nm and 0.312 nm, corresponding to the (104) plane of BiFeO_3_ and the (130) plane of BiVO_4_, were also observed by high-resolution transmission electron microscopy (HR-TEM) (Figure 3d). The establishment of a nanoscale heterostructure was confirmed by the unique interface arising from their close contact (Figure 3e). Subsequently, X-ray photoelectron spectroscopy and Raman spectroscopy were performed on BiFeO_3_, BiVO_4_ and the BiFeO_3_/BiVO_4_ perovskite structure, further indicating the successful establishment of a p-n heterojunction between BiFeO_3_ and BiVO_4_ and the changes in valence states, and along with the reallocation of electrical charge from BiVO_4_ to BiFeO_3_, the catalytic capacity for CO_2_ and N_2_ directional adsorption was further enhanced, which in turn contributed to the enhancement of electrocatalytic capacity and reaction [42,43]. The electrochemical impedance spectrometry (EIS) demonstrated that the construction of local charges in the rationally developed BiFeO_3_/BiVO_4_ catalyst was conducive to the specific adsorption and activation of molecules by taking advantage of their electrophilic character as well as their nucleophilic properties [44,45]. In other words, the chemical adsorption of the N_2_ molecules onto BiFeO_3_/BiVO_4_ hybrids initiated the C–N coupling reaction, while the partial charge redistribution, devoted to the full exposure of active sites and the acceleration of electrocatalytic dynamics, facilitated the arrangement of C–N bonds as well as the production of required *NCON* intermediates [15,46].

#### 2.2.2. Mott–Schottky Heterostructure Bi-BiVO_4_

Despite the morphology and elemental distribution of the Bi-BiVO_4_ hybrids being quite similar to that of pristine BiVO_4_, there are clear exclusive heterostructural interfaces between the metallic Bi and BiVO_4_. Once such heterojunctions are formed, the space charge region was exhibited via self-driven charge transfer from BiVO_4_ to Bi, generating local electrophilic and nucleophilic regions. The Bi-BiVO_4_ hybrids also generated a greater electrochemical active surface area (ECSA) than pristine BiVO_4_, indicating that the designed space charge region is favorable for exposing more dynamic sites. The electrochemical impedance spectra of Bi-BiVO_4_ hybrids revealed shorter semicircular and steeper slopes compared to the pristine BiVO_4_ sample, confirming that coupling of Bi-BiVO_4_ hybrids to spatial charge zones promoted effective charge transfer and regendered the surface of Bi and BiVO_4_ locally nucleophilic and electrophilic active [45,47,48]. 

**Figure 4:**
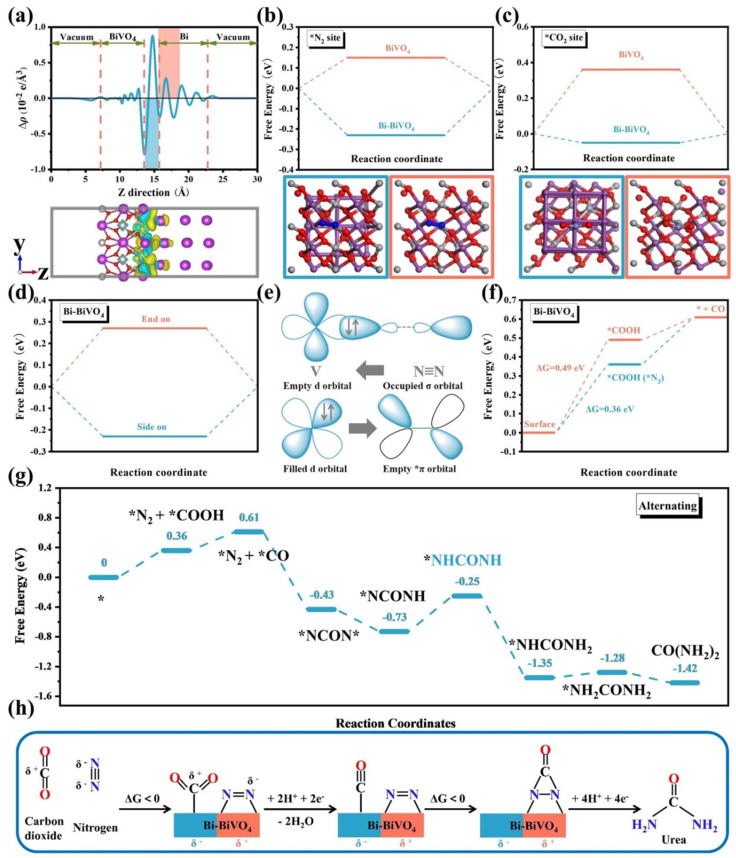
(**a**) Top: Planar average charge density difference along the z-direction for the Bi-BiVO4 heterojunction; bottom: charge density difference of the Bi-BiVO_4_ heterojunction, and yellow and cyan indicate electron accumulation and depletion, respectively, with isosurface values of 0.002 e Å^−3^. Free energy diagrams for (**b**) N_2_ and (**c**) CO_2_ adsorption on BiVO_4_ and Bi-BiVO_4_, the bottom figures are the corresponding calculation models; (**d**) N_2_ adsorbed on the Bi-BiVO_4_ by different configurations; (**e**) simplified schematic of N_2_ bonding to V center; (**f**) free energy diagrams for CO_2_ reduction with and without N_2_ adsorption onto Bi-BiVO_4_ hybrids; (**g**) electrolytic urea production via the alternating mechanism; (**h**) mechanism of the electrocatalytic urea synthesis based on synergistic effects of the Bi-BiVO_4_ Mott–Schottky heterostructure. Reproduced with permission from Ref. [8]. (* represent the active sites; The blue color represent Bi, while orange color represent BiVO4) Copyright 2021 John Wiley and Sons.

C atoms in CO_2_ are partially positively charged and electrophile, while N atoms in N_2_ are partially negatively charged and nucleophilic [49,50]. As revealed by Bader charge analysis (Figure 4a), in the Bi-BiVO_4_ hybrids, the interface between the metallic Bi and BiVO_4_ materials stimulates spontaneous charge transfer and creates a spatial charge region that drives the target surface reaction. As revealed by the Bader charge analysis (Figure 4a), the interface between the metallic Bi and BiVO_4_ materials promoted spontaneous charge transfer and created a spatial charge region that drives the target surface reaction. The space–charge region played a decisive role in promoting gas adsorption because the original BiVO_4_ model exhibited a much higher adsorption energy for N_2_ and CO_2_ than the Bi-BiVO_4_ hybrid model (Figure 4b,c). At the interface, the bending band promoted charge redistribution until the electrocatalyst achieved thermal equilibrium. The formation of the unique space–charge region was also observed by X-ray photoelectron spectroscopy, where oppositely charged regions occurred at heterogeneous interfaces, altering electron density around the interface and promoting the adsorption of target small molecules and consecutive proton processes (Figure 4h) [51].

Detailed analysis confirmed that the interface of the novel heterostructure possessed local charge regions and harnessed the unique “back-donation” mechanism to achieve specific adsorption and activation (Figure 4e). Of these, N_2_ binding at the local electrophilic BiVO_4_ region can be further activated by energy-optimized side-on configurations (Figure 4d). Moreover, the catalysts effectively avoided the poison of CO and accelerated the generation of exothermic *NNH intermediate (Figure 4g), thus facilitating the efficient process of the reaction. The mechanism involves the initial adsorption of both CO_2_ and N_2_ on the catalyst surface, followed by the conversion of CO_2_ to CO and the activation of N_2_ to N=N intermediates. These intermediates then undergo coupling to produce the desired *NCON* urea precursor, which is subsequently hydrogenated to yield the urea product.

### 2.3. Frustrated Lewis Pairs (FLPs)

#### 2.3.1. InOOH Nanoparticles 

Electrocatalytic synthesis is regarded as a sustainable and efficient way to produce urea. Nevertheless, this strategy is severely hindered by several intrinsic limitations, including weak chemisorption [52,53,54], poor activation efficiency [55], and the formidable challenge of C–N bond formation [8,15].

**Figure 5:**
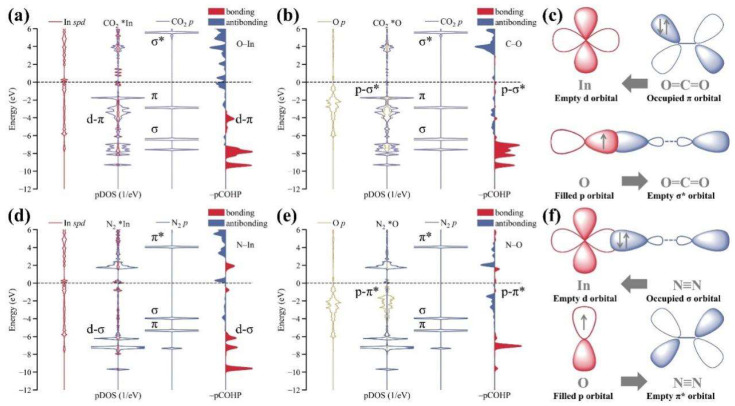
The activation of N_2_ and CO_2_ on the FLP sites (A and B). The pDOS of CO_2_ molecule adsorbed at (**a**) the Lewis acidic In site and (**b**) the Lewis base O site (in the OH group) in artificial FLPs and the pCOHP for (**a**) In–O interactions and (**b**) C–O interactions (right) during the gas molecules’ activation by artificial FLPs. (**c**) The schematic illustration of charge donation–acceptance process between the FLP and the CO_2_ molecule. (**d**,**e**) The pDOS of N_2_ the molecule adsorbed at (**d**) the Lewis acidic In site and (**e**) Lewis base O site (in the OH group) in artificial FLPs and the pCOHP for (**d**) In–N interactions and (**e**) O–N interactions (right) during the gas molecules’ activation by artificial FLPs. (**f**) The schematic illustration of charge donation–acceptance process between FLP and N_2_ molecule. Reproduced with permission from Ref. (* represent the active sites) [59]. Copyright 2022 Elsevier.

Frustrated Lewis acid–base pairs (FLPs) formed by Lewis acids (LAs) and Lewis bases (LBs) that do not bond with each other [56,57,58]. Nevertheless, the electron-deficient type of the acidic In site of InOOH and the electron-rich type of the alkaline In-OH can complete the synergistic activation effect by interbinding with the bonding and anti-bonding orbitals of the reactants, thus progressively achieving the specific adsorption of CO_2_ and N_2_ and supplying a strategic scheme for solving the major problem of C–N coupling [8,15,59,60,61,62]. The bonding orbital activation mechanisms for each of the two gas molecules are shown in the diagram (Figure 5), i.e., the CO_2_ activation mechanism, d-π + p-σ*, and the N_2_ activation mechanism, d-σ + p-π* [59].

#### 2.3.2. Flower-Like Ni_3_(BO_3_)_2_ Nanocrystals 

**Figure 6:**
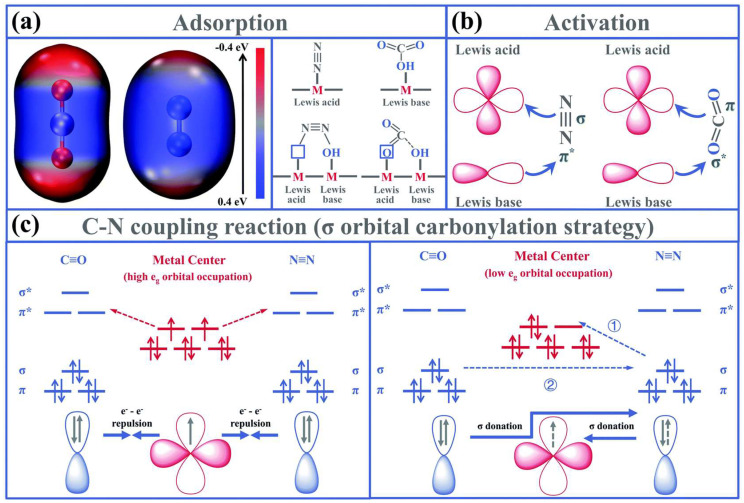
Schematic illustration of the contribution of artificial frustrated Lewis pairs in the (**a**) adsorption, (**b**) activation and (**c**) C–N coupling reaction steps during the urea electrosynthesis process. Reproduced with permission from Ref. (* represent the active sites) [6]. Copyright 2021 Royal Society of Chemistry.

In flower-like Ni_3_(BO_3_)_2_ nanocrystals, nickel (Ni) sites with lower e_g_ orbital acted as Lewis acids (LAs) and the adjacent surface hydroxyl groups served as Lewis bases (LBs), which integrated together form frustrated Lewis pair (FLP)-active sites. In Figure 6a, the surface electrostatic potential analysis showed that the C atom in CO_2_ lacked electrons, whereas the N atom in N_2_ was enriched with electrons. When the inert molecules proceeded over FLPs, the detailed activation mechanisms were listed below: the π orbital of CO_2_ first donated electrons to the empty d orbital of the metal site in the LA, while the empty σ* orbital of CO_2_ received electrons from the electron-rich LB sites (Figure 6b). Moreover, when the metal sites with low LA occupancy participated in the reaction, the preferentially adsorbed *N=N* initially donated their σ orbital electrons to the metal sites of LA. Subsequently, the carbine characteristic *CO directly coupled with *N=N* through the transfer of σ orbital electrons, generating *NCON* urea precursors (Figure 6c). Thus, the effective adsorption, activation and C–N bond coupling of N_2_ can be achieved. The empty orbitals of LA and the lone pair orbitals of the LB would, respectively, engage with the bonding and antibonding orbitals of CO_2_ and N_2_, inducing the polarization of the gas molecules and cleavage of chemical bonds. As a result, a collaborative activation effect would arise.

## 3. Mechanism for Breaking Chemical Bonds and Directional Coupling: Achieving C–N Bond Coupling

Regarding the current electrocatalytic urea synthesis process, achieving efficient C–N coupling remains an active and comparatively difficult area of academic research. CO_2_ and N_2_, once preferentially adsorbed onto the catalyst, face intense competing reactions, such as the generation of the by-product NH_3_ and the inevitable hydrogen evolution reaction at the cathode [4,11]. Therefore, developing catalysts that can effectively lower the energy required for CO_2_- and N-containing raw materials is a paramount issue [22,63]. This section primarily reviews several new frontier catalysts in terms of the theoretical prediction of catalysts, defective catalysts and frustrated Lewis pairs and their utilization to promote efficient urea production through different principles such as transforming *O groups into *OH groups to facilitate intermediate coupling [18], utilizing oxygen vacancies to enhance coupling efficiency [64] and utilizing frustrated Lewis pair properties to promote the C–N coupling reaction [6,59].

### 3.1. Theoretical Prediction of Catalysts

#### 3.1.1. Double Transition-Metal MXenes

**Figure 7:**
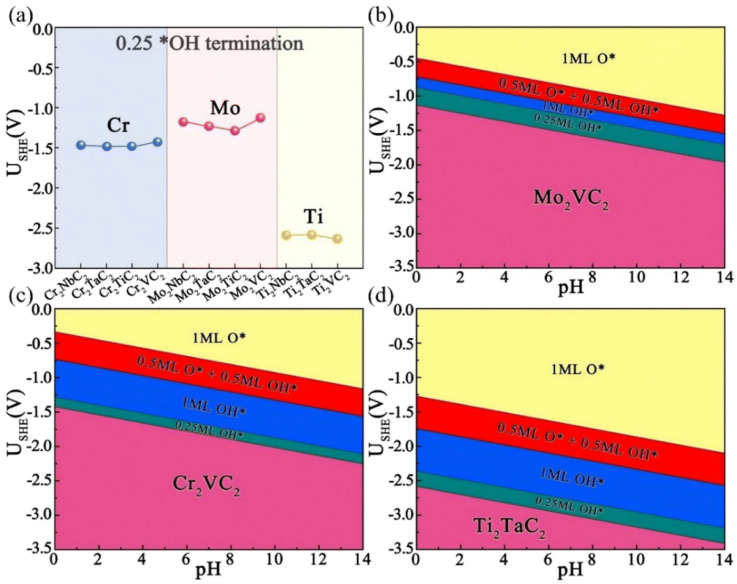
(**a**) USHE of different MXenes under 0.25 OH* termination at pH = 0 and surface Pourbaix diagrams of (**b**) Mo_2_VC_2_, (**c**) Cr_2_VC_2_ and (**d**) Ti_2_TaC_2_. Reproduced with permission from Ref. [32]. (* represent the active sites; Different color represent different concentration of mediates including *O and *OH) Copyright 2023 Royal Society of Chemistry.

The molecular–catalyst interaction mechanism in MXenes is described in Section 2.1.1, and the results show that the surfaces of MXenes with exposed activation sites were more favorable for electrocatalyzed urea synthesis. The Pourbaix diagram of the discovered bimetallic surface of Mxenes was calculated in order to establish the equilibrium potential and pH at the most stable configuration of the molecule [65]. The charge of the *O groups on the surface of MXenes on an energetically acidic surface habitually changed to *OH groups as the potential of the outer electrode was increased, whereas the surface of the catalyst exhibited an exposed trend. The emergence of these exposed active sites offers enormous feasibility for coupling the intermediate *CO with *N_2_, *NNH and *NHNH (Figure 7) [32].

#### 3.1.2. Conductive MOF Co–PMDA–2-mbIM (PMDA = pyromellitic dianhydride; 2-mbIM = 2-methyl benzimidazole)

Electrocatalytic C–N coupling is a pivotal step in the electrocatalytic synthesis of urea which determines the selectivity of the reaction [8,15]. If C–N coupling is not possible, only CO and NH_3_ by-products can be formed. In contrast, once the C–N coupling reaction has occurred, the desired urea product can be generated. However, similar orbitals of *N=N* and *CO intermediates will result in electrostatic interactions between them and thus prevent the spontaneous coupling of the reaction. Conductive metal–organic frameworks (c-MOFs) can accomplish the regulation of electron orbitals through a multiplicity of mechanisms, offering new opportunities for the coupling of C–N bonds [66,67,68,69,70]. As reported, the high-occupied e_g_ orbitals are known to be unable to accommodate intermediate electrons to the detriment of the C–N coupling process, while the low-occupied e_g_ orbitals can trigger a “σ orbital carbonylation” process which facilitates the spontaneous C–N coupling; therefore, the novel proof-of-concept catalyst conductive MOF-Co-PMDA-2mbIM was developed [71].

**Figure 8:**
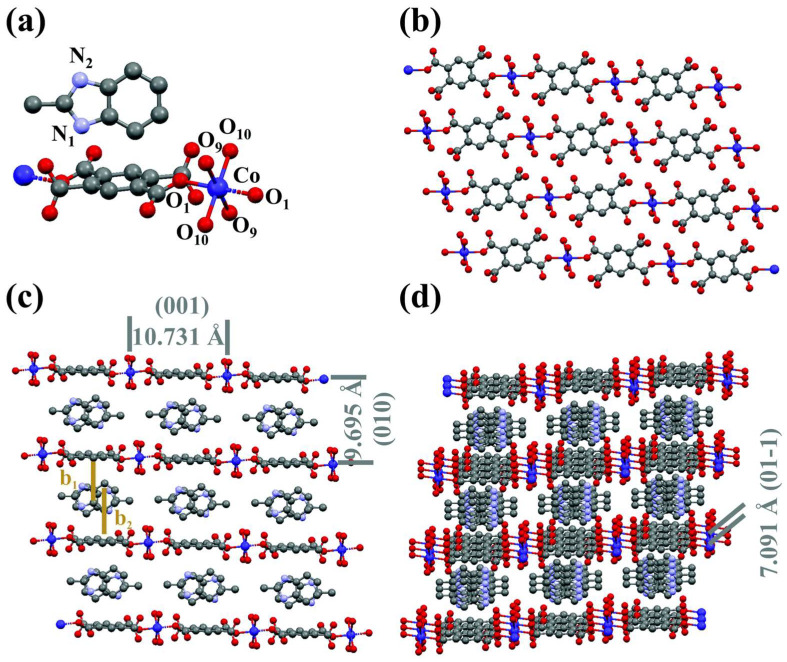
Structural diagrams of Co–PMDA–2-mbIM: (**a**) the coordination structure of Co metal sites; (**b**) the 2D layer structure fabricated from 1D chains; (**c**) the 3D intercalation structure topology; and (**d**) a 3D intercalation structure perspective view. Reproduced with permission from Ref. [71]. (Different color represent different atoms. Blue, grey, purple, red represent Co, C, N and O atoms) Copyright 2022 RSC Publishing.

When the organic pyromellitic dianhydride (PMDA) and 2-methyl benzimidazole (2-mbIM) utilized as the host and the guest, it will accelerate the electron transfer rate and optimize charge transfer through host–guest interactions (Figure 8). Experiments have demonstrated that the spontaneous electron transfer was achieved by the host–guest interaction mechanism, which induced the generation of the electrophilic region of Co-PMDA and the nucleophilic region of 2-mbIM and further realize the targeted adsorption and activation of the reactants. The investigations also proved that the host–guest interaction optimized the filling of e_g_ electrons and realized the transition of high Co^3+^ (HS: t^4^_2g_e^2^_g_) to low spin states Co^4+^ (IS: t^4^_2g_e^1^_g_) [71,72,73,74,75,76]. Once the low e_g_ orbital center Co^4+^ (IS: t^4^_2g_e^1^_g_) accepted σ electrons from the intermediate *N=N* orbital, *CO readily coupled with the *N=N* via electron transfer from the σ orbital, enabling the coupling of the C–N bond, which in turn generated an important intermediate *NCON*, enabling the efficient urea synthesis process.

### 3.2. Defective Catalysts

#### 3.2.1. Oxygen Vacancy-Enriched CeO_2_

Defect engineering has been elucidated as a promising strategy for achieving efficient electrocatalytic reactions [77,78], and the yield rate of urea synthesis can be significantly enhanced by introducing oxygen vacancies (Vos). This investigation demonstrated that CeO_2_ enriched in oxygen vacancies served as an compelling electrocatalyst to balance the central transitional of *NO by embedding it into additional sites, thus facilitating consecutive C–N coupling rather proton coupling reactions [22]. Consequently, the introduction of Vo is also able to boost the adsorption of the reaction molecules by constructing coordinatively unsaturated sites [64]. 

**Figure 9:**
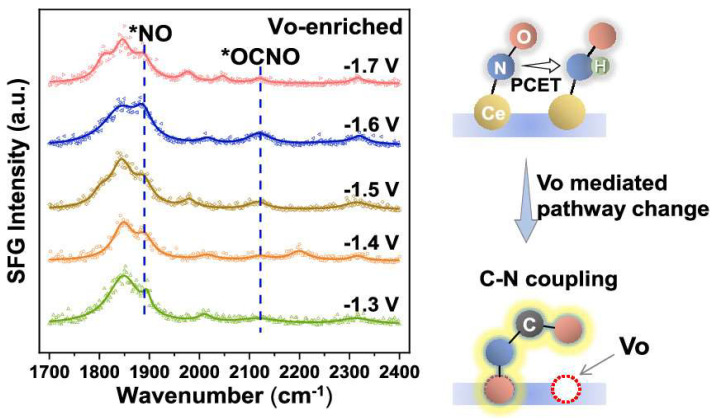
New oxygen vacancy-enriched CeO_2_ workflow. Reproduced with permission from Ref. [64]. (* represent the active sites) Copyright 2022 American Chemical Society.

The establishment of Vo determined its construction of allocation-indurated sites and enhanced the adsorption of the reactant. With the presence of Vo, the adsorption of CO_2_ can be significantly reduced by correlating the CO_2_ yield and oxygen vacancy-enriched CeO_2_ [79,80]. And the corresponding reaction mechanism is that Vo stabilizes nitrogen-containing intermediates and inhibits their hydrogenation, thus expediting the C–N coupling as well as following urea generation. Electrocatalytic product analysis confirmed that *OCNO intermediates are associated with the formation of urea, and pre-adsorbed oxygen atoms cause the relative amount of *OCNO to increase, shifting the dominant vibrational band of *OCNO (Figure 9), where the peak position is slightly lower than that of the reported *NCO intermediates [81]. It was originally shown that Vo promotes the production of *OCNO by stabilizing the *NO intermediates and thus improves the synthetic performance of urea [64]. Impressively, the urea yield of the Vo-CeO_2_ catalyst was nearly three times higher than that of pure CeO_2_ and even better than some of the reported noble metal catalysts.

#### 3.2.2. PdCu-TiO_2_

Deficient catalysts have already contributed astonishingly to diverse chemical fields as well as bringing new opportunities to the field of the electrocatalytic synthesis of urea [82]. TiO_2_ nanosheets are considered to be marvelous vehicles for the preparation of catalysts due to their tendency to generate oxygen vacancies (OVs) when undergoing reduction reactions at elevated temperatures [83]. TiO_2_-PdCu alloy nanoparticles were fabricated through the co-reduction in metal precursors. The rationally designed catalysts possess bimetallic synergistic effects and an optimized electronic structure, providing a new opportunity to move toward efficient C–N coupling reactions [4,82]. 

**Figure 10:**
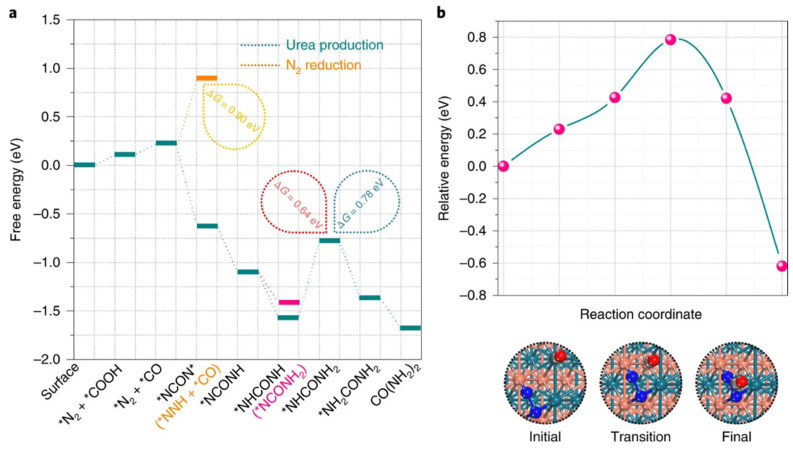
(**a**) Free energy diagram of urea production. (**b**) The reaction pathway of *NCON* formation. The structures of the initial, transition and final states along with the *NCON* formation are presented. The green, orange, blue, red and grey balls represent Pd, Cu, N, O and C atoms, respectively. Reproduced with permission from Ref. [4]. (* represent the active sites) Copyright 2020 Springer Nature.

Relevant experiments demonstrated that alloy structures substantially enhanced the adsorption capacity of N_2,_, that the electronic interactions of the bimetal completed the benign competitive reaction between CO_2_ and N_2_ and that oxygen vacancies (OVs) were introduced to improve the activation capacity of Pd_1_Cu_1_/TiO_2_ for the reactants [4]. The thermodynamically spontaneous coupling reaction of *N=N* with CO drives the formation of the C–N bond, which in turn facilitated the production of the urea product. The theoretical calculation further revealed that the inert N_2_ molecule can be absorbed onto the catalyst surface via the side-on configuration, which rendered the d orbital electron readily transferable from metal sites to the π* orbital of N_2_, and the bond energy between N and N was thus broken [84,85]. Moreover, the conversion process of CO_2_RR to CO was also facilitated by the activation of N_2_ at the adjacent sites. From the corresponding free energy diagram, the CO_2_RR readily generated the CO products with the pre-adsorption of the N_2_ molecule (Figure 10a). Once the CO was released, *N=N* easily bonded with CO to form the urea precursor *NCON* (Figure 10b). During the subsequent N hydrogenation, the carbonyl group also played an influential role and the stability of *NCONH was well maintained [86,87].

## 4. Discussion

Given the limited product variety of electrocatalytic CO_2_ reduction reactions, expanding the range of products by introducing additional reactants that provide elemental diversity is necessary. Electrocatalytic urea synthesis using CO_2_- and nitrogen-containing compounds as feedstock is an energy-efficient and environmentally friendly alternative to industrial urea synthesis processes. In this review, the design and working principles of existing catalysts are analyzed to expand the scope of electrocatalytic applications for the synergistic conversion of CO_2_ and N_2_ for urea synthesis by analyzing the catalyst interaction mechanisms and chemical bond-breaking and directional coupling mechanisms (Table 1). Future in-depth studies are needed regarding the following aspects:

(1) Compared to CO_2_, the adsorption and activation of inert and non-polar N_2_ molecules is significantly problematic when CO_2_ and N_2_ are utilized as the feedstock for urea electrosynthesis. Hence, when designing catalysts, priority should be given to enhancing the catalytic activity of N_2_. The investigation of nitrogen reduction reactions and electrocatalytic urea synthesis should complement each other.
(2) To obtain more insight into the electrocatalytic process, the advanced in situ operational characterization can contribute to analyzing the chemical and electronic structures of the catalytic sites, as well as the essential intermediates and the critical steps in the catalytic reaction. Theoretical calculations can be applied to optimize the electrocatalytic behavior of the catalyst. Furthermore, in situ operational characterization can uncover the exact conformational relationships of the catalyst by monitoring the structural transformation of the active site during the electrocatalytic process.
(3) Multiphase catalytic processes occur at the two- or three-phase interface, and the adsorption, dissolution and diffusion of intermediates as well as the generated products governed by the composition of the electrodes and membranes take place. Simultaneously, diffusion is dramatically impacted by the structure of the electrode and membrane assembly in the catalyst layer. Fluid dynamics can be controlled by optimizing the equipment structure, e.g., by tweaking the batch flow rate. The specificity of the products of the cascade reaction can be adjusted by controlling the fluid dynamics, including the mass velocity and volume pressure of the gas and liquid phases. Meanwhile, the selectivity of cascade reaction can be modulated by evaluating the device structure. A proper electrocatalytic device design can effectively regulate the product selectivity, stability and energy efficiency of electrocatalytic reactions, representing a robust tool for high-performance devices that can achieve superior urea electrosynthesis performance.
(4) Molecular–catalyst interaction mechanisms as well as chemical bond breakage and directional coupling mechanisms have explained the mechanism of electrocatalytic synthesis of urea from different microscopic perspectives, contributing to solving the difficult problems of adsorption, activation and the coupling of reactant molecules in urea synthesis. Meanwhile, this mechanism could be applied in other directions, such as the molecular catalyst interaction mechanisms in electrocatalytic nitrogen reduction, which could enhance the electrophilicity of catalyst surfaces through the carrier effect and inhibit proton reduction. Similarly, the chemical bond breaking and directional coupling mechanisms enhance the efficiency of ammonia synthesis by accelerating the breaking of chemical bonds through electron feeding. Furthermore, this mechanism will be of considerable interest in other areas.

## 5. Conclusions

In summary, this review summarizes the specific adsorption principles and promotion of the C–N coupling reaction for urea production through two microscopic mechanisms, i.e., molecule–catalyst interactions and chemical bond-directed coupling mechanisms. In the light of the current frontier of scientific research development, the catalysts in emerging fields, such as bimetallic catalysts, MXenes, frustrated Lewis pairs, heterostructures, etc., and the corresponding catalytic mechanism toward urea synthesis, were also introduced. This paper provides new research perspectives while summarizing the previous research in order to improve the efficiency of the electrocatalytic synthesis of urea. Regrettably, this review may not list all the typical catalysts and some novel catalysts currently under research and development are not presented in great detail. Nevertheless, the detailed analysis and summary of the two micro-mechanisms in this review should be helpful for a wider audience to quickly grasp the remaining problems and challenges in the electrobond-directed catalytic synthesis of urea.

## Figures and Tables

**Table 1:** The relationship between the catalysts and synthesis methods as well as the corresponding electrocatalytic activity for urea synthesis.

Catalysts	Synthesis Methods	Faraday Efficiency/Yield Rate
MXenes [27]	Theoretical calculations	--
Pd–Cu [31]	Wet chemistry impregnation + carbonization fixation process	69.1 ± 3.8%
BiFeO_3_/BiVO_4_ [13]	Hydrothermal method	17.18%
Bi-BiVO_4_ [8]	Hydrothermal method + NaBH_4_ reduction	12.55%
InOOH [53]	Hydrothermal method + annealing treatment	20.97%
Ni_3_(BO_3_)_2_ [6]	Wet chemistry + low-temperature annealing	20.36%
Co–PMDA–2-mbIM [65]	Hydrothermal method	48.97%
CeO_2_ [58]	Hydrothermal method + annealing treatment	943.6 mg h^−1^ g^−1^
PdCu-TiO_2_ [4]	High temperature reduction	8.92%
